# Utilization of modern contraceptive methods among women of reproductive age in Senegal: A multilevel mixed-effects analysis

**DOI:** 10.1371/journal.pone.0323899

**Published:** 2025-05-20

**Authors:** Hiwot Tezera Endale, Habtu Kifle Negash, Winta Tesfaye, Fethiya Seid Hasen, Tseganesh Asefa, Destaye Tirite Gelaw, Yihun Tefera Ayenew, Nebebe Demis Baykemagn, Tirualem Zeleke Yehuala, Mihret Getnet

**Affiliations:** 1 Department of Medical Biochemistry, School of Medicine, College of Medicine and Health Sciences, University of Gondar, Gondar, Ethiopia; 2 Department of Human Anatomy, School of Medicine, College of Medicine and Health Sciences, University of Gondar, Gondar, Ethiopia; 3 Department of Epidemiology and Biostatistics, Institute of Public Health, College of Medicine and Health Sciences, University of Gondar, Gondar, Ethiopia; 4 Department of Human Physiology, School of Medicine, College of Medicine and Health Sciences, University of Gondar, Gondar, Ethiopia; 5 Department of Medical Nursing, School of Nursing, College of Medicine and Health Sciences, University of Gondar, Gondar, Ethiopia; 6 Department of Health Informatics, Institute of Public Health, College of Medicine and Health Sciences, University of Gondar, Gondar, Ethiopia; Debre Tabor University, ETHIOPIA

## Abstract

**Background:**

Despite high fertility rates in low- and middle-income countries, modern contraceptive use remains low. In Senegal, there is limited use of modern contraceptives, and few studies have explored the factors influencing their adoption. This study aims to identify the determinants of modern contraceptive use among women of reproductive age in Senegal.

**Method:**

Data from the 2023 Senegal Demographic and Health Survey, covering 8,833 women, were analyzed using a multilevel logistic regression model to identify factors associated with modern contraceptive use. Adjusted odds ratios (AORs) with 95% confidence intervals (CIs) were used, and statistical significance was set at p < 0.05. Analysis was conducted using STATA version 17.

**Results:**

The utilization of modern contraceptives among reproductive-aged women in Senegal was 32.16% (95% CI: 31.19, 33.15). Significant factors associated with its use included age (25–34: AOR = 1.46, 35–44: AOR = 1.59, > 44: AOR = 1.72), secondary education (AOR = 1.19), employment (AOR = 1.38), being undecided about having more children (AOR = 1.39), not wanting more children (AOR = 2.42), age at first sex (≥18: AOR = 1.16), and first birth (≥19: AOR = 0.65). Other factors include being visited by a field worker (AOR = 1.32), visiting a health facility (AOR = 2.04), media exposure (AOR = 1.42), higher household income (middle: AOR = 1.23, rich: AOR = 1.38), and having one or more births (one: AOR = 2.06, two or more: AOR = 1.72). Women living in rural areas (AOR = 0.75) or in regions such as Ziguinchor (AOR = 0.60), Diourbel (AOR = 0.50), Tambacounda (AOR = 0.27), Louga (AOR = 0.61), Matam (AOR = 0.37), Kédougou (AOR = 0.64), and Sédhiou (AOR = 0.67) were less likely to use contraceptives.

**Conclusion:**

Factors such as age, education, employment, media exposure, and healthcare access are critical determinants of modern contraceptive use in Senegal. Women in rural areas and specific regions face barriers to contraceptive use. Targeted interventions in these areas could enhance family planning and reproductive health outcomes in Senegal.

## Introduction

Family planning offers numerous benefits, including a significant reduction in maternal and infant mortality rates [1–3]. Contraceptives are an integral part of family planning services. According to the World Health Organization (WHO), modern contraceptive methods include condoms, injectables, pills, sterilization, intrauterine devices (IUDs), implants, and emergency contraception. These methods play a crucial role in maintaining women’s reproductive health and preventing unintended pregnancies [4]. However, due to method-related challenges such as side effects and difficulties in consistent use or access, 85% of women become pregnant within one year of discontinuing contraception [5].

Globally, 65% of women of reproductive age use modern contraceptive methods, whereas sub-Saharan Africa has a much lower rate of 29.6% [6]. This low prevalence contributes to higher rates of unintended pregnancies, unsafe abortions, and maternal mortality in the region. Enhancing family planning services is essential to address these issues and improve reproductive health outcomes [7–10].

Senegal is among the African countries with a significantly high fertility rate and rapid population expansion [11,12]. In Senegal, the total fertility rate (TFR) varies, with rural areas having an average of 6.35 children per woman, while urban areas have a lower rate of 3.9 children per woman. The country also has one of the lowest family planning usage rates, at just 26.3%, according to the 2017 Continuous Demographic and Health Survey (C-DHS) [11]. A significant proportion of women in Senegal are not receiving family planning services, with an unmet need estimated at 29%. This means that about one in every three married women who wish to stop having children or delay their next pregnancy are not using contraception. The unmet need is even higher in the Dakar region, where it stands at 32%, even though 25% of the country’s population resides in this area [13].

Different factors affect the use of family planning services in sub-Saharan Africa. In Nigeria, factors such as age, parity, and partner disapproval play key roles in the use of family planning services [14,15]. Similarly, in Malawi, the uptake of modern contraceptive methods is influenced by factors such as wealth quintile, educational level, and fertility intentions [16]. While in Ethiopia, significant determinants include socioeconomic status, urban or rural location, and regional disparities [17].

In Senegal, although contraceptive use remains low, there has been limited research on the factors contributing to this issue [11,18–21]. Existing studies on contraceptive use primarily rely on data from the 2012/13 DHS [20], the 1992–2014 DHS [19] and the 2017 CDHS [11]. These studies often provide fragmented insights into contraceptive use, focusing either on single factors or specific geographic area [18,21,22]. However, none of these studies have investigated the link between individual level factors such as socioeconomic status and fertility intentions and community-level factors, such as healthcare access and regional disparities and contraceptive use in Senegal. Moreover, while studies in other sub-Saharan African countries, such as Nigeria, Malawi, and Ethiopia, have identified key determinants of contraceptive use [14–17], similar research has not been done in Senegal. Existing studies have failed to comprehensively address the complex interplay between individual and community-level factors in shaping contraceptive use. This gap in the literature highlights the need for a more nuanced and up to date analysis of the determinants of modern contraceptive use in Senegal, which could inform more effective policies and interventions aimed at improving reproductive health outcomes.

There is a significant lack of up-to-date, nationally representative data on modern contraceptive use and its determinants among women of reproductive age. Therefore, this research will contribute to a more detailed understanding of the factors influencing contraceptive use, thereby supporting the development of targeted interventions to address the unmet need for family planning services in Senegal. Furthermore, this study seeks to fill this gap by evaluating modern contraceptive use and its determinants using the latest national data, offering valuable insights for informed policy development and interventions in reproductive health in Senegal.

## Methods and materials

### Study design

We used a cross-sectional study design based on data from the SDHS 2023.

### Data source and study setting

This study utilized secondary data from the 2023 Senegal Demographic and Health Survey (DHS), conducted between February and August of that year. The survey was nationally representative and employed a two-stage stratified sampling process designed by the Demographic and Health Survey Authority. Data were collected from 14 regions across the country, covering a wide range of public health topics and involving 8,423 households. A subset of 9,008 women of reproductive age (15–49 years) was analyzed. These women provided detailed information on their use of modern contraceptives, forming the basis of the investigation into contraceptive use patterns.

### Study variables and measurements

#### Outcome variable.

The outcome variable for this study is the utilization of modern contraceptive methods. It is categorized as ‘Yes’ for individuals who report using any of the following modern methods: female sterilization, male sterilization, pills, intrauterine devices (IUDs), injectables, implants, male condoms, female condoms, emergency contraception, the Standard Days Method (SDM), the Lactational Amenorrhea Method (LAM), or other recognized modern contraceptive methods. Conversely, individuals are categorized as ‘No’ if they use traditional methods such as rhythm (calendar method) or withdrawal, or if they do not use any family planning method. This binary classification allows for the examination of factors influencing modern contraceptive use versus non-modern or no contraceptive use [9,11,23,24].

#### Explanatory variables.

Explanatory variables were selected at both individual and community levels based on their demonstrated association with the outcome variable, as identified through a review of relevant literature, and their availability in the DHS datasets [11,21,25–33]. At the individual level, these variables included age, women’s education, husband’s education, modern contraceptive use, women’s occupation, number of living children, fertility preference, age at first sexual intercourse, age at first birth, contact with health extension workers, visits to health facilities, perceived distance to health facilities, marital status, access to media (If a person has access to one of the information sources radio, TV, or newspapers that individual considered as having access to media), wealth index (“poorest” and “poorer” categories recoded as “poor”; “middle” remains unchanged; and “richer” and “richest” categories recoded as “rich”), births within the last five years, history of terminated pregnancies, decision-making autonomy, religion, and source of contraceptive methods. At the community level, the variables considered were place of residence (urban or rural), region, and distance to the nearest health facility. This combination of individual and community-level factors provides a comprehensive framework for understanding the determinants of modern contraceptive utilization.

### Data management and analysis

Data handling and analysis were performed using STATA version 17. Before analysis, the presence of the outcome variable in the SDHS dataset was confirmed, and all study variables were checked for missing data. Records with missing observations were excluded from the analysis to maintain data quality. The dataset was weighted to account for sample non-representativeness across regions in Senegal, ensuring accurate estimates and standard errors. The final weighted sample consisted of 8,833 reproductive-age women. Descriptive analysis was conducted using percentages to summarize the study variables. Due to the hierarchical nature of DHS data, which includes multiple levels such as clusters, households, and individuals, advanced methods like multilevel modeling (MLM) are essential to address intra-cluster correlation and variability at each level. In this analysis, multilevel logistic regression was employed to identify key factors associated with modern contraceptive use. Four models were tested: The Null Model, serving as a baseline with no predictors; Model II, which includes individual-level variables to assess their impact; Model III, focusing on community-level variables to examine their influence; and Model IV, combining both individual and community-level variables to understand their combined effects. This methodology provides a more accurate understanding of relationships at each level while adjusting for intra-cluster correlation. Significant factors were reported using Adjusted Odds Ratios (AORs) with 95% Confidence Intervals (CIs) and a p-value < 0.05. Four models were constructed to estimate both the fixed effects of individual and community-level factors, as well as the random effects to capture between-cluster variability. To compare and assess model fit, several statistical metrics were applied, including the Intra-class Correlation Coefficient (ICC), Likelihood Ratio (LR) test, Median Odds Ratio (MOR), and deviance (-2LLR). Multicollinearity was checked using the Variance Inflation Factor (VIF), with a result of 1.47, indicating no significant multicollinearity [30,34,35].

### Ethics approval and consent to participants

Permission to access the data was obtained from the Measure DHS Program via an online request at the DHS Program. The publicly available dataset contains no personal identifiers, so no additional ethics approval was required. We registered on the DHS website, requested the dataset, and received authorization to download the data files, complying with all relevant guidelines and regulations.

## Result

### Characteristics of the study participants

This analysis included data from 8833 weighted reproductive-age women. The majority were aged 25–34 years (37.69%), and over half (52.59%) had no formal education, while 58.07% of their husbands also lacked formal education. Regarding employment, 58.60% of women were not working, and most resided in rural areas (54.18%). 38.83% and 40.98% of the participants were classified as poor and rich respectively, though 88.66% reported having access to media. Reproductive history showed that 55.46% of women had their first sexual encounter after turning the age of 18, and 58.49% gave birth after turning 19. A majority (76.90%) expressed a desire for more children, with 37.81% reporting having one to two children. Furthermore, 79.07% of the participants had not experienced a terminated pregnancy. Accessibility to healthcare services was varied, with 37.16% identifying distance to health facilities as a major challenge, and 83.87% reporting no visits from health extension workers. The prevalence of modern contraceptive use among reproductive-age women was 32.16% (95% CI: 31.19, 33.15). Regional variations were observed, with the highest prevalence reported in Dakar (23.33%) and the lowest in Kedougou (1.07%). Women who accessed private healthcare providers (55.02%), were Married & living with a partner (89.95%), or participated in household decision-making (35.44%) exhibited higher prevalence rates (Table 1).

**Table 1:** Characteristic of the study participants.

Variables	Categories	Weighted Frequency	Percentage
Age	15–24	2316.91	26.23
	25–34	3,328.88	37.69
	35–44	2,673.48	28.49
	>44	513.46	5.38
Women education	No education	4,645.29	52.59
	Primary	1,956.79	22.15
	Secondary	1,884.88	21.34
	Higher	345.79	3.91
Husband education	No education	4613.75	58.07
	Primary	970.39	12.21
	Secondary	863	10.87
	Higher	556.97	7.01
	Don’t know	940.51	11.84
Modern contraceptive use	No	5991.81	67.84
	yes	2,840.93	32.16
Women occupation	Not working	5,176.06	58.60
	Working	3,656.68	41.40
Place of Residence	Urban	4047.53	45.82
	Rural	4785.21	54.18
Region	Dakar	2,060.59	23.33
	Ziguinchor	280.35	3.17
	Diourbel	907.23	10.27
	Saint-Louis	700.37	7.93
	Tambacounda	422.55	4.78
	Kaolack	643.81	7.29
	Thiès	1,316.03	14.90
	Louga	504.23	5.71
	Fatick	492.89	5.58
	Kolda	399.69	4.53
	Matam	338.15	3.83
	Kaffrine	407.28	4.61
	Kédougou	94.11	1.07
	Sédhiou	265.46	3.01
Number of living child	None	889.75	10.07
	1–2	3,339.97	37.81
	3–4	2,492.11	28.21
	>4	2,110.92	23.90
Fertility preference	Want other	6,792.25	76.90
	Undecided	387.55	4.39
	want no more	1,652.94	18.71
Age at first sex	<18	3,934.36	44.54
	>=18	4,898.38	55.46
Age at first birth	<19	3,666.03	41.51
	>=19	5,166.71	58.49
Visited by health extension worker	No	7,407.87	83.87
	Yes	1,424.88	16.13
Visited Health facilities	No	2,677.52	30.31
	Yes	6,155.23	69.69
Distance to health facility	Big problem	3,282.55	37.16
	Not big problem	5,550.19	62.84
Marital status	Never married	348.55	3.95
	Married & living with partner	7,945.25	89.95
	Widowed, divorce & separated	538.94	6.10
Access to media	No	1,002.07	11.34
	Yes	7,830.68	88.66
Wealth index	Poor	3,429.86	38.83
	Middle	1,783.49	20.19
	Rich	3,619.39	40.98
Birth in the last five years	None	2,432.84	27.54
	One	4,190.32	47.44
	Two and plus	2,209.59	25.02
Terminated pregnancy	No	6,983.86	79.07
	Yes	1,848.89	20.93
Decision making	No	5,129.43	64.56
	Yes	2,815.81	35.44
Religion	Muslim	8,603.57	97.41
	Christian	228.48	2.59
	Animist	0.69	0.01
Source of contraceptive	Government	2,548.66	28.85
	Private	4,859.92	55.02
	Don’t know	1,424.16	16.12

### The random effect analysis

Table 2 summarizes the multilevel logistic model of both individual and community-level variables. 12.73% variance in modern contraceptive utilization was observed among women of reproductive age across clusters as shown in the ICC of the null model. MOR in the null model indicated heterogeneity in modern contraceptive prevalence across clusters, with a MOR value of 1.94 (95% CI = 1.80, 2.1), which implied that the odds of utilization of modern contraceptive among reproductive age women in clusters with higher prevalence was 1.94 times greater compared to those in clusters with lower levels of modern contraceptive use. This showed that the existence of significant heterogeneity in modern contraceptive utilization across different clusters. Model III was identified as the best-fitting model, as it demonstrated the lowest deviance among all the models tested. The proportion change in the variance (PCV) in this model was 49.98% which showed that 49.98% of community variance observed in the null model was explained by both community and individual level variables.

**Table 2:** Random-effect logit models in predicting modern contraceptive utilization among reproductive age women in Senegal.

Parameters	Null model	Model I	Model II	Model III
Cluster level Variance (SE)	0.48(0.06)	0.38(0.05)	0.21(0.03)	0.24(0.04)
ICC	12.73%	10.2	6.1	6.80
PCV	Reference	21.74%	55.56%	49.98%
MOR	1.94 (95% CI = 1.80, 2.10)	0.97(95% CI = 0.74, 1.28)	1.550 (95% CI = 1.45, 1.67)	1.26 (95% CI = 1.18, 1.38)
LLR	−5307.269	−4404.557	−5214.784	−4354.333
Deviance	10,614.538	8,809.114	10,429.568	8,708.666

SE = standard error, ICC = intra-class correlation coefficient, PCV = proportional change in variance, MOR = median odds ratio, LLR = log-likelihood ratio

### Fixed effect analysis

Women aged 25–34, 35–44 and >44 were 1.46 (95% CI = 1.26, 1.69), 1.59 (95% CI = 1.34, 1.90), 1.72 (95% CI = 1.29, 2.28) times more likely to utilize modern contraceptive methods compared to women aged 15–24 respectively. Additionally, women who had completed secondary education were 1.19 times more likely to use modern contraceptives compared to those without formal education (AOR = 1.19, 95% CI = 1.03, 1.40). Regarding employment status, working women had a 1.38 times higher likelihood (AOR = 1.38, 95% CI = 1.22, 1.55) of using modern contraception compared to women without occupation. Regarding fertility preferences, women who were undecided about having more children or those who did not wish for additional children were inclined to utilize modern contraceptive compared to women who wanted more children with AOR of 1.39 (95% CI = 1.08, 1.81) and 2.42 (95% CI = 2.08, 2.83) respectively.

Women who had sexual intercourse after the age of 18 (≥18) were 1.16 times more likely to use modern contraceptives compared to those who experienced sex before the age of 18 (AOR = 1.16, 95% CI = 1.01, 1.32). On the other hand, women who gave birth at the age of 19 or older had a 35% decreased likelihood of utilizing modern contraceptives compared to their counterparts who gave birth at a younger age (AOR = 0.65, 95% CI = 0.57, 0.74). Women who were visited by a field worker were 1.32 times more likely to utilize modern contraceptives compared to those who were not visited (AOR = 1.32, 95% CI = 1.14, 1.53). Similarly, women who visited a health facility had a 2.04 times higher likelihood of using modern contraceptives compared to those who did not (AOR = 2.04, 95% CI = 1.79, 2.31). Furthermore, women with household media exposure were 1.42 times more likely to use modern contraceptives than those without media access (AOR = 1.42, 95% CI = 1.17, 1.72).

Women from middle-income households were 1.23 times more likely (AOR = 1.23, 95% CI = 1.03, 1.46), and those from rich households were 1.38 times more likely (AOR = 1.38, 95% CI = 1.13, 1.68) to utilize modern contraceptive methods compared to women from poor households. Additionally, women who had given birth once in the last five years were 2.06 times more likely (AOR = 2.06, 95% CI = 1.77, 2.39), and those who had given birth twice or more were 1.72 times more likely (AOR = 1.72, 95% CI = 1.45, 2.04) to use modern contraceptives compared to women who had not given birth in the last five years. Women residing in rural areas of Senegal were 25% less likely to utilize modern contraceptive methods than their urban counterparts. Moreover, women living in the regions of Ziguinchor, Diourbel, Tambacounda, Louga, Matam, Kédougou, and Sédhiou were 40%, 50%, 73%, 39%, 63%, 36%, and 33% less likely, respectively, to use modern contraceptive methods compared to those residing in Dakar (Table 3).

**Table 3:** Multilevel multivariable logistic regression for determinants of modern contraceptive use among reproductive age women in Senegal.

Categories	Model I	Model II	Model III
Age			
15–24	1	–	1
25–34	1.45(1.25, 1.68)^**^	–	1.46(1.26, 1.69)^**^
35–44	1.58(1.33, 1.89)^**^	–	1.59(1.34,1.90)^**^
>44	1.71(1.28, 2.27)^**^	–	1.72(1.29, 2.28)^**^
Women Education			
No education	1	–	1
Primary	1.16(1.01, 1.34)^*^	–	1.14(0.99, 1.32)
Secondary	1.21(1.04, 1.42)^*^	–	1.19(1.03, 1.40)^*^
Higher	1.14(0.81, 1.61)	–	1.11(0.79, 1.57)
Women occupation			
Not working	1	–	1
Working	1.43(1.27, 1.61)^**^	–	1.38(1.22, 1.55)^**^
Fertility preference			
Want more	1	–	1
Undecided	1.41(1.09, 1.82)^*^	–	1.39(1.08, 1.81)^*^
Want no more	2.43(2.08, 2.83)^**^	–	2.42(2.08, 2.83)^**^
Age at first sex			
<18	1	–	1
>=18	1.19(1.04, 1.36)^*^	–	1.16(1.01, 1.32)^*^
Age at first birth			
<19	1	–	1
>=19	0.65(0.57, 0.74)^**^	–	0.65(0.57, 0.74)^**^
Visited by field worker			
No	1	–	1
Yes	1.33(1.15, 1.53)^**^	–	1.32(1.14, 1.53)^**^
Visited health facility			
No	1	–	1
Yes	2.11(1.87,2.39)^**^	–	2.04(1.79, 2.31)^**^
Household media exposure			
No	1	–	1
Yes	1.53(1.27, 1.86)^**^	–	1.42(1.17, 1.72)^**^
Wealth index			
Poor	1	–	1
Middle	1.34(1.14, 1.58)^**^	–	1.23(1.03, 1.46)^*^
Rich	1.69(1.42, 2.00)^**^	–	1.38(1.13, 1.68)^*^
Birth in the last five years			
None	1	–	1
One birth	2.02(1.74, 2.35)^**^	–	2.06(1.77, 2.39)^**^
Two and plus	1.71(1.44, 2.02)^**^	–	1.72(1.45, 2.04)^**^
Terminated pregnancy			
No	1	–	1
Yes	0.92(0.81, 1.06)	–	0.91(0.79, 1.04)
Decision making		–	
No		–	
Yes	1.05(0.93, 1.18)	–	1.05(0.93, 1.18)
Residence			
Urban	–	1	1
Rural	–	0.60(0.52, 0.69)^**^	0.75 (0.63, 0.91)^*^
Distance to health facility			
Not big problem	–	1	1
Big problem	–	0.94(0.84, 1.05)	0.98(0.87,1.12)
Regions			
Dakar	–	1	1
Ziguinchor	–	0.73(0.51, 1.04)	0.60(0.39, 0.92)^*^
Diourbel	–	0.55(0.39, 0.78)^*^	0.50(0.35, 0.74)^**^
Saint-Louis	–	0.93(0.67, 1.28)	1.04(0.73,1.49)
Tambacounda	–	0.24(0.17, 035)^**^	0.27(0.18, 0.41)^**^
Kaolack	–	0.83(0.59, 1.17)	0.81(0.56, 1.17)
Thiès	–	0.97(0.71, 1.31)	0.85(0.61, 1.19)
Louga	–	0.53(0.38, 0.74)^**^	0.61(0.42, 0.88)^*^
Fatick	–	0.96(0.68, 1.33)	0.94(0.65, 1.36)
Kolda	–	0.75(0.54, 1.06)	0.81(0.55, 1.18)
Matam	–	0.31(0.22, 0.45)^**^	0.37(0.24, 0.55)^**^
Kaffrine	–	0.93(0.67, 1.30)	1.02(0.71, 1.49)
Kédougou	–	0.59(0.42, 0.86)^*^	0.64(0.42, 0.96)^*^
Sédhiou	–	0.63(0.44, 0.89)^*^	0.67(0.45, 0.99)^*^

Significant at

** p* < *0.001,

* p* < *0.05

## Discussion

Reducing maternal morbidity and mortality and achieving universal health coverage, including access to essential healthcare services in sub-Saharan Africa (SSA) by 2030, are key targets of the third Sustainable Development Goal (SDG) [36,37]. Providing women of reproductive age with the ability to make informed decisions about their fertility such as determining the timing and number of their children is essential for upholding women’s rights, promoting gender equality, and supporting societal advancement. Modern contraceptives are instrumental in preventing maternal, neonatal, and infant morbidity and mortality.

This study examines the prevalence of modern contraceptive use among reproductive-age women, based on data from the 2023 Senegal demographic health (SDHS). Among 8,833 weighted respondents, the prevalence of modern contraceptive method use was 32.16% (95% CI: 31.19–33.15). Our findings align with studies conducted in Ethiopia, Kenya and Ghana [9,38–40]. The observed prevalence in this study is higher than reported in previous studies conducted in Senegal, Sierra Leone, Uganda, Ghana, Burkina Faso, and Mali [11,41–44]. However, it remains lower compared to findings from studies conducted in South Africa and Kenya [40,45,46]. The discrepancy might be associated with the differences in the study population, setting, varying levels of awareness regarding modern contraceptive methods and the time gap between the studies, all of which can significantly influence modern contraceptive method utilization.

Women’s age, level of education, occupation, desire for more children, age at first sexual encounter, age at first birth, wealth index, visits from field workers, health facility visits, media exposure, childbirth within the last five years, place of residence, and region were associated with modern contraceptive modern use in Senegal.

Our findings indicated that the use of modern contraceptives increases with age. This aligns with previous studies [47] and contrasts with findings from studies conducted in Ghana [32], Ethiopia [23], Mali [48] and Uganda [49]. The differences could be due to variations in sample size, study design, settings, and study populations. Young girls may experience challenges in accessing healthcare services, especially reproductive health services, because of limited knowledge about contraceptives, where to access contraceptive services and financial barriers. Additionally, some younger women may not be sexually active.

In our study, women with secondary education were 1.19 times more likely to use modern contraceptives compared to those with no formal education aligns with findings from studies conducted in in Ethiopia [25,50,51], Haiti [52], Ghana [32], Uganda [53], Cameroon [31] and Senegal [11]. This consistency highlights the critical role education plays in influencing contraceptive use across diverse contexts. Educated women are often better equipped to access and understand health information, which empowers them to make informed decisions about their reproductive health. Education enhances autonomy, enabling women to advocate for their health needs and seek healthcare services proactively. Furthermore, education expands economic opportunities, which can influence fertility preferences by allowing women to prioritize career and personal goals alongside family planning [26,30,54].

The finding that employed women have better utilization of modern contraceptives compared to non-employed women is consistent with studies conducted in Ethiopia [55], Malawi [56], Zambia [57], Cameroon [31], Indonesia [58], Bangladesh [59] and India [60]. This trend can be attributed to several interrelated factors. Employment often provides women with greater exposure to education and a wider social network. Through workplace interactions and professional environments, employed women may gain access to diverse sources of information, including health-related resources, which can increase their awareness and understanding of modern contraceptive methods. Furthermore, employment fosters financial independence and decision-making autonomy, which can enhance their ability to seek and utilize healthcare services, including family planning.

Women who planned to have more children were less likely to use modern contraceptives, consistent with findings from previous studies on modern contraceptive utilization [31,61–63]. This may stem from the perception of children as a valuable resource, contributing to household economic growth by participating in the labor force [64,65].

This study found a significant link between age at first sexual intercourse and the use of modern contraceptives. Women who began sexual activity at age 18 or older were more likely to use modern contraceptives than those who started before 18. This aligns with previous research [66] but contradicts the findings of studies [67,68]. This suggests that delaying sexual initiation may be associated with better reproductive health outcomes, potentially due to increased awareness, autonomy, and access to contraceptive methods. The results underscore the importance of sexual health education and empowerment in enhancing contraceptive use.

Women who gave birth at age 19 or older were 35% less likely to use modern contraceptives compared to those who had children at a younger age, a finding that contrasts with a study from Zambia [57]. This variation could be attributed to differences in cultural norms, social structures, and economic conditions across the study populations. In some contexts, older mothers may perceive a lower risk of unintended pregnancies or may have already reached their desired family size, reducing the perceived need for modern contraceptives. Furthermore, these women might face barriers such as limited access to family planning services or a preference for traditional contraceptive methods. Variations in reproductive health policies, healthcare systems, and contraceptive availability may also play a role in these differing outcomes.

In our study, women who received visits from a field worker were more likely to use modern contraceptives compared to those who did not, a finding consistent with studies conducted in Ethiopia [17,69,70] and Malawi [71]. However, this contrasts with a study from Zambia, where field worker visits were not significantly associated with contraceptive use [72]. This difference could be attributed to variations in healthcare systems across countries. Furthermore, women who visited a health facility had a higher likelihood of using modern contraceptives, a result that aligns with findings from a study in Sierra Leone [41]. The study also revealed a positive association between media exposure and modern contraceptive use. Women who were exposed to media were more likely to use modern contraceptive methods compared to those who were not, consistent with findings from other studies [33,43,55,73–76]. This may be because mass media serves as a powerful tool for increasing awareness, providing information about the benefits and availability of modern contraceptives, and motivating women to adopt these methods.

In this study, women from poor-income households were less likely to utilize modern contraceptive methods compared to those from middle and rich income households. This finding is consistent with previous studies conducted in Malawi [47], Ethiopia [23] and Nigeria [77], but contrasts with findings from Ghana [32]. The observed disparity in outcomes may be attributed to differences in socioeconomic contexts, access to healthcare services, cultural norms, or variations in family planning policies and programs across these countries. Women who had given birth in the last five years been more likely to use modern contraceptives compared to those who had not, a finding that contradicts studies [33,55]. This disparity may be attributed to differences in study populations or contexts. In some settings, recent childbirth may prompt women to seek family planning services to space or limit future pregnancies, while in other contexts, cultural norms or limited access to postpartum contraceptive services might reduce contraceptive use among new mothers. Additionally, healthcare systems and the availability of postpartum family planning programs could influence these differing outcomes.

Women living in rural areas of Senegal were 25% less likely to use modern contraceptive methods compared to their urban counterparts, a finding consistent with other studies [11,19,57,78]. This disparity may be attributed to lower education levels in rural areas, which can limit awareness of family planning options. Additionally, rural women often face greater challenges in accessing healthcare services and contraceptives due to geographic and infrastructural barriers. Cultural norms in rural settings may also favor larger family sizes, reducing the perceived need for modern contraceptive methods.

Women living in the regions of Ziguinchor, Diourbel, Tambacounda, Louga, Matam, Kédougou, and Sédhiou were significantly less likely to use modern contraceptive methods compared to those in Dakar, with reduced likelihoods of 40%, 50%, 73%, 39%, 63%, 36%, and 33%, respectively. These regional disparities in contraceptive use are consistent with previous studies [11,19], which identified lower use of modern contraceptives in almost all regions compared to Dakar. This variation can be attributed to the concentration of family planning services and a greater number of health facilities in urban areas like Dakar [18]. Previous studies have also highlighted the unequal distribution of healthcare infrastructure and personnel across regions, with a concentration of resources in urban areas, particularly in Dakar, which has contributed to disparities in contraceptive use [18–20,79].

## Strength and limitation of the study

This study provides valuable insights into modern contraceptive use by utilizing extensive national survey data. DHS data is designed to be nationally representative, allowing for the generalization of findings to the wider population. With its large sample size, the study examined contraceptive use at both individual and community levels, enabling the exploration of hierarchical or clustered influences on outcomes. However, its cross-sectional design limits the ability to establish causal relationships, as it only captures data at a single point in time. Additionally, the reliance on secondary data means that critical factors such as service quality and cultural influences were not considered, potentially omitting key aspects that may influence contraceptive use. The use of self-reported data introduces the possibility of recall bias, as participants may not accurately report their contraceptive use, which could affect the validity of the findings. Furthermore, the exclusion of other factors, such as policy changes or personal experiences over time, limits the comprehensiveness of the analysis.

## Conclusion

Modern contraceptive utilization was moderate in this study, with a prevalence of 32.16%. Despite its critical role in improving reproductive health outcomes, utilization remains suboptimal, particularly in rural and economically disadvantaged regions. Factors such as higher education, employment, and media exposure were associated with increased usage, while rural residence, lower wealth status, and certain regions exhibited significantly lower rates. Additionally, disparities were influenced by age, fertility preferences, age at first sexual encounter, age at first birth, health facility visits, field worker outreach, recent childbirth, and place of residence. Targeted interventions should focus on improving access to family planning services and raising awareness in rural and underserved areas, particularly among women with limited education and lower socioeconomic status. Educational campaigns and healthcare outreach must address these gaps while promoting equitable access to reproductive health services. Strengthening healthcare systems and ensuring the equitable distribution of family planning resources across regions are vital to achieving the Sustainable Development Goals, particularly in reducing maternal morbidity and mortality and promoting gender equality. Future research should explore cultural and systemic barriers to contraceptive use to design effective, context-specific strategies.

## Recommendation

Based on our finding, targeted interventions, such as mobile clinics in rural areas should be implemented to increase access to contraceptive services where they are most needed. Radio campaigns in local languages could also help raise awareness and change attitudes toward modern contraception, especially in communities with limited literacy [80,81]. Drawing inspiration from successful programs like Ethiopia’s Health Extension Workers, who have played a key role in improving access to reproductive health services in rural areas [70,82,83], Senegal could strengthen community-based health worker programs to provide counseling, education, and service delivery at the grassroots level. These programs would improve access to reliable information and support for contraceptive use, ultimately enhancing family planning outcomes in Senegal. Moreover, it is recommended to conduct primary study to gain a more thorough understanding of contraceptive use in Senegal.

## Abbreviations

DHS: Demographic and Health Survey
IUD: Intrauterine Device
SDHS: Senegal Demographic and Health Survey.
